# Still a Long Way to Go. Editorial for the Special Issue “Understanding Autism Spectrum Disorder”

**DOI:** 10.3390/brainsci11081062

**Published:** 2021-08-13

**Authors:** Eugenio Aguglia, Laura Fusar-Poli

**Affiliations:** Department of Clinical and Experimental Medicine, Psychiatry Unit, University of Catania, Via Santa Sofia 78, 95123 Catania, Italy; eugenio.aguglia@unict.it

Although many years have passed since the first descriptions of autism spectrum disorder (ASD) [1,2], the scientific community is still far from having a thorough knowledge of the epidemiology, etiopathology, phenotypical presentation, and potential therapies of ASD. The 17 papers included in the Special Issue “Understanding autism spectrum disorder” fully reflect the complexity and heterogeneity of this condition. Through both data-driven approaches and literature reviews, this collection aims to provide novel insights for clinical practice and future research.

Epidemiological investigations suggest that the prevalence of ASD has considerably increased over the last decades. Recent studies have estimated that around 2% of the general population might be on the autism spectrum [3,4]. Both genetic and environmental factors seem to contribute to the etiopathology of the autistic condition [5]. Mutations of SHANK3—a gene crucial to the neurobehavioral phenotype—are among the most studied genetic alterations. Moreover, there is evidence that non-neuronal processes, such as oxidative stress and mitochondrial dysfunctions, may be implicated in ASD etiology [6]. In this Special Issue, Kartawy et al. analyzed the cortical tissues of ASD mouse models and found that SHANK3 mutations may induce aberrant protein S-nitrosylation [7]. This abnormal mechanism may in turn favor mitochondria-related processes, including oxidative phosphorylation, oxidative stress, and apoptosis, potentially contributing to ASD pathoetiology [7]. These findings may further support the need for a paradigm shift, as suggested by other researchers [5].

The increased ASD prevalence has led to a considerable burden on national health systems [8]. Leveraging data from a large international sample [9,10], Bieleninik and Gold aimed to estimate the components and costs of standard care for autistic children in Europe, suggesting that services receipt is widely variable across different countries, and that costs for the management of ASD are generally remarkable [11]. Interestingly, costs appeared related to participants’ age and intellectual functioning [11]. Indeed, it should be noted that ASD is frequently accompanied by psychiatric [12] as well as medical comorbidities [13] across the lifespan. Given the burden on health services, the importance of early diagnosis appears intuitive, in order to improve the long-term outcome [14]. In the Special Issue “Understanding autism spectrum disorder”, Akter et al. [15] proposed a transfer learning-based face recognition framework to favor the early detection of children with ASD.

If, on one hand, great emphasis has been given to early diagnosis, on the other hand, the broadening of diagnostic criteria has led to the search for the “lost generation” of autistic adults [16,17,18]. Particular attention has been recently paid to the so-called “female autism phenotype”, a slightly different presentation of autistic symptoms not completely explained by the current diagnostic criteria or recognized by common standardized instruments, typically based on male features [19]. In this regard, Rynkiewicz et al. [20] have proposed a critical reflection on the importance of sex-formulated questions in the Polish version of the Social Communication Questionnaire (SCQ), a screening tool for ASD. Moreover, Gesi et al. [21] discussed potential gender differences in misdiagnosis and delayed diagnosis of adults with ASD who have no language impairments or intellectual disability. In line with another recent study [18], the authors reported that autistic women show a significantly greater delay in referral to mental health services and higher age at diagnosis than autistic men [21].

Another relevant topic that emerged in the Special Issue is the role played by subthreshold autistic traits in the onset and presentation of psychopathological conditions. Dell’Osso et al. [22] investigated the relationship between autistic traits, ruminative thinking, and suicidality in a clinical sample of subjects with bipolar disorder and borderline personality disorder. Additionally, Concerto et al. [23] showed that both autistic traits and attention deficit-hyperactivity disorder symptoms could predict the severity of Internet Gaming Disorder in a group of adult videogame players.

The phenomenology and clinical implications of restrictive and repetitive behaviors (RRB), sensory issues, and perception appeared to be of particular interest for researchers. First, Keller et al. [24] mapped the literature on RRB in animal models of ASD, showing how a phylogenic approach could be useful in disentangling the complex thematic of stereotypies. Second, Grossi et al. [25] attempted to classify different RRB patterns after video-recording a group of autistic individuals within a naturalistic context. Third, Jamioł-Milc et al. [26] investigated the relationship between tactile stimuli and quality of sleep in ASD. Fourth, Kadlaskar et al. [27] compared different aspects of tactile cues between autistic and typically developing children, further underlying the importance of research on sensory processing in this population. Finally, Damiani et al. reviewed the literature concerning source monitoring, a crucial skill for self–other distinction and integration between internally- and externally-generated experiences [28].

It is worth mentioning that the different way of thinking and behaving of autistic individuals described in the aforementioned studies does not necessarily represent a weakness. In fact, special talents and unsuspected strengths are frequently found in autistic individuals, regardless of their functioning level and symptoms severity [29]. In this Special Issue, Solazzo et al. [30] measured the emergence of early hyperlexic traits in a group of young children with ASD. According to the authors, the early ability to name and recognize letters and numbers was associated with a higher level of RRB, but also more promising social skills and language abilities [30]. In their pilot study, Brondino et al. [31] reported that a group of people with severe ASD did not show a higher susceptibility for COVID-19 infection and severity of the disease compared with a neurotypical group. The authors hypothesized that ASD may represent a protective factor against COVID-19 infection due to the pro-inflammatory status observed [32,33].

One of the most critical and debated topics in this research field remains the development of an effective therapy for autistic core and associated symptoms. Indeed, even if psychosocial interventions should represent the first-line treatments, off-label medications are frequently prescribed in clinical practice [34]. A systematic review included in this Special Issue provided evidence that LEGO^®^-based therapy may improve social interaction in children with ASD [35]. The heterogeneity of the autistic condition and the adoption of sparse outcome measures in clinical trials undoubtedly represent obstacles for finding an effective therapy [36]. The Clinical Global Impression (CGI) scales are among the most common and easily interpretable tools used in clinical trials investigating treatments for ASD [36]. In this collection, Siafis et al. [37] implemented a method to estimate the number of treatment responders from the CGI-Improvement scores. This technique may help harmonize the results of clinical trials and pool them in meta-analyses to better understand the actual efficacy of treatments for ASD. Finally, Bertamini et al. [38] developed a novel observational coding system to quantify the child–therapist interaction in ASD intervention. The authors suggested that increased synchrony, successful strategies in engaging the children and longer and more complex interchanges, may be associated with favorable outcomes [38].

In summary, the Special Issue “Understanding autism spectrum disorder” represents a rich collection of papers that may help professionals improve their knowledge about this somehow enigmatic condition. Nevertheless, this collection also raises a number of questions and provides intriguing cues to encourage clinicians and researchers to keep investigating the fascinating world of ASD.
